# Case report: Going through pregnancy safely after twice partial nephrectomy for bilateral kidneys with HLRCC-associated RCC

**DOI:** 10.3389/fonc.2022.932996

**Published:** 2022-10-18

**Authors:** Kunhan Dai, Wencheng Jiang, Siyu Chen, Shengjun Luo, Siwei Ding, Delin Wang

**Affiliations:** Department of Urology, The First Affiliated Hospital of Chongqing Medical University, Chongqing, China

**Keywords:** pregnancy, hereditary carcinoma, renal cell carcinoma, partial nephrectomy, FH

## Abstract

**Background:**

HLRCC-associated RCC (hereditary leiomyomatosis and renal cell cancer-associated renal cell carcinoma) caused by germline mutations of the fumarate hydratase (*FH*) gene is a rare autosomal dominant genetic renal cancer. At present, there are no reports of bilateral kidneys with HLRCC-associated RCC, let alone safe pregnancy after twice partial nephrectomy for bilateral kidney HLRCC-associated RCC.

**Case presentation:**

We report a 25-year-old woman with bilateral renal tumors detected by ultrasound screening during a routine checkup. CT revealed a soft tissue mass in the parenchyma of the left kidney and a nodular soft tissue mass in the lower pole of the right kidney. She underwent robot-assisted laparoscopic left partial nephrectomy and underwent laparoscopic right partial nephrectomy 3 months after the first surgery. Heterozygous mutation in the *FH* gene on the patient’s tumor tissue was detected by genetic testing. Combined with the patient’s medical history, microstructure and immunohistochemical staining of tumor tissue, and genetic test results, the pathological reports after two operations concluded HLRCC-associated RCC. Then, she was injected with interferon and nivolumab as a preventative treatment against tumor recurrence. Up to 38 months after surgery, having given birth to a baby, till now there was no tumor progression.

**Conclusions:**

This is a clinically significant case, as it provides a reference for pregnancy in patients undergoing partial nephrectomy for bilateral kidneys with HLRCC-associated RCC and may indicate an effective approach to preventing tumor recurrence by nivolumab in patients with HLRCC-associated RCC.

## Introduction

Hereditary leiomyomatosis and renal cell carcinoma (HLRCC) syndrome, also known as Reed syndrome, is an autosomal dominant genetic syndrome caused by germline mutation of the fumarate hydratase (*FH*) gene and characterized by early-onset multiple uterine fibroids, cutaneous leiomyomas, and renal cell carcinoma (1, 2). Only a few hundred families around the world have been reported to have the syndrome. However, because the disease is not completely overt, there may be patients with HLRCC syndrome who have not been diagnosed definitely (3). HLRCC-associated RCC (hereditary leiomyomatosis and renal cell cancer-associated renal cell carcinoma), also known as *FH*-deficient RCC (fumarate hydratase-deficient renal cell carcinoma) caused by the *FH* gene, is a highly malignant renal cell carcinoma that can rapidly metastasize throughout lymph nodes and systemic organs. The lung is the most common site of metastasis, and the patient usually has a poor prognosis (4). Muller et al. tracked the disease progression of 182 patients with HLRCC from 114 families in France from 2004 to 2016; 19% had a history of renal cell carcinoma, of which 82% had metastasized at the time of diagnosis or within the following 3 years and the median survival of patients with metastatic cancer was 18 months (5). Cases of bilateral kidneys with HLRCC-associated RCC are extremely rare, and there are no reports of pregnancy after partial nephrectomy for bilateral kidneys with HLRCC-associated RCC. Therefore, clinicians are short of clinical experience for women of childbearing age with HLRCC-associated RCC, so that the aim of this article is to provide a reference for pregnancy for women of childbearing age with HLRCC-associated RCC.

We herein report a female patient with bilateral HLRCC-associated RCC. After twice partial nephrectomy, she went through pregnancy and gave birth to her baby safely.

## Case report

**Figure 1:**
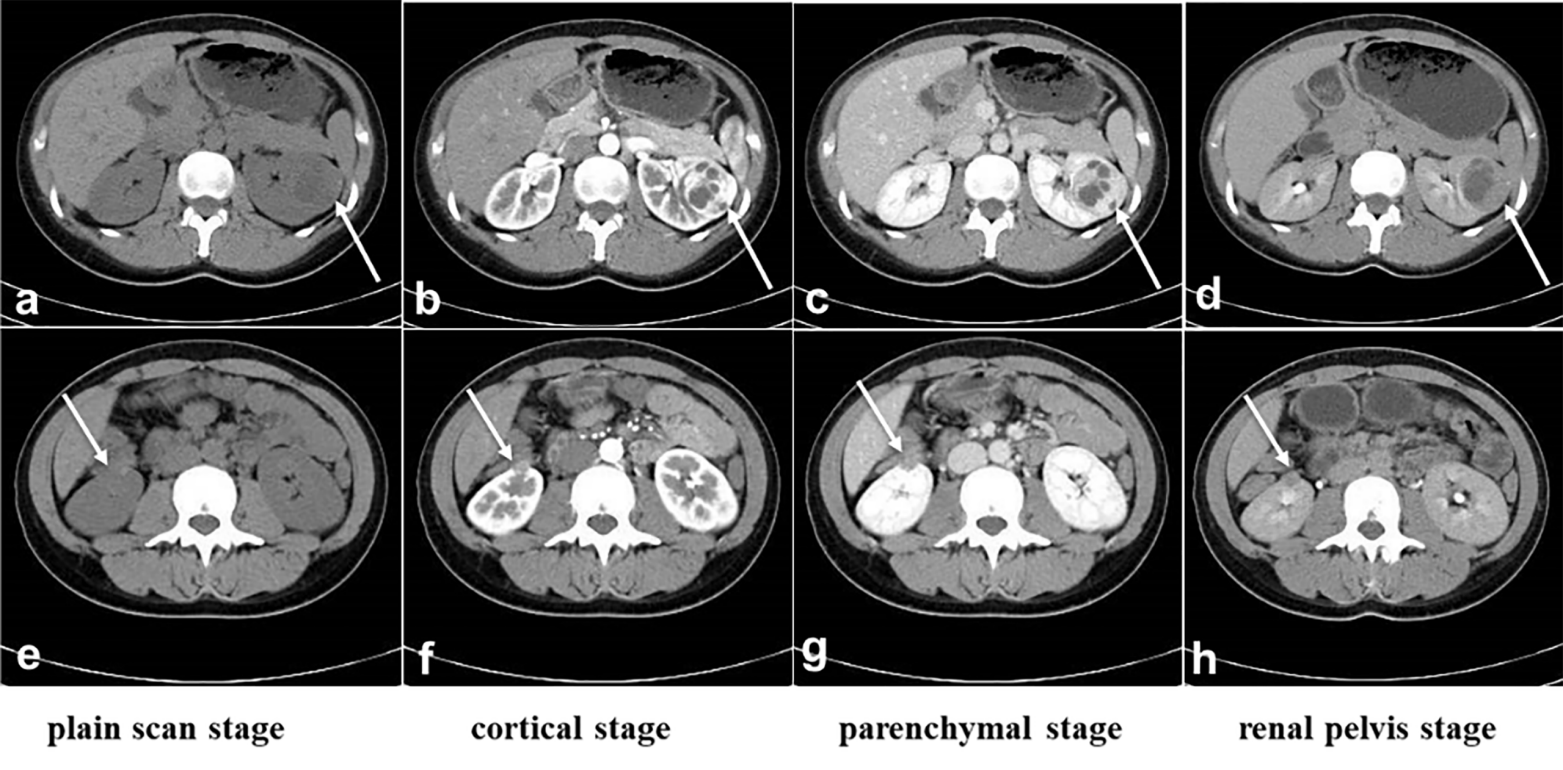
CT before surgery. **(A-D)**: The location and size of the tumor in the left kidney. **(E, F)**: The location and size of the tumor in the right kidney. **(A)** and **(E)**: Plain scan stage, **(B, F)**: cortical stage, **(C, G)**: parenchymal stage, **(D, H)**: renal pelvis stage. The white arrow indicates the tumors.

**Figure 2:**
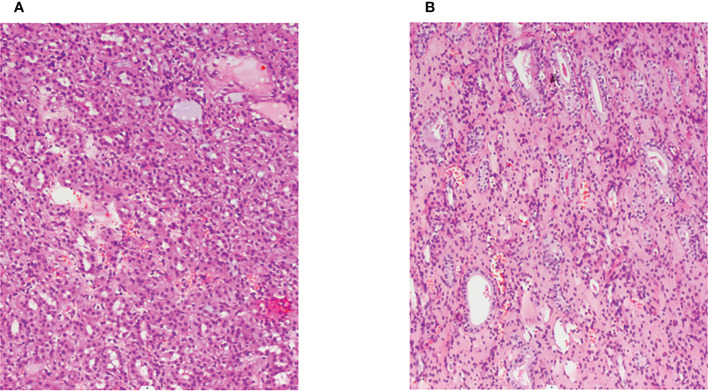
Pathological section (H&E, ×100). **(A, B)**: Hematoxylin–eosin staining showed that the cytoplasm of renal cell carcinoma was eosinophilic and had a tubular and glandular structure.

**Figure 3:**
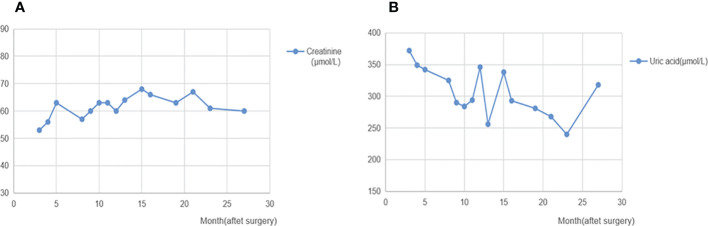
Creatinine and uric acid after surgery. **(A)**: Changes of creatinine after surgery. **(B)**: Changes of uric acid after surgery. The levels of creatinine and uric acid after surgery follow-up were in the normal range.

**Figure 4:**
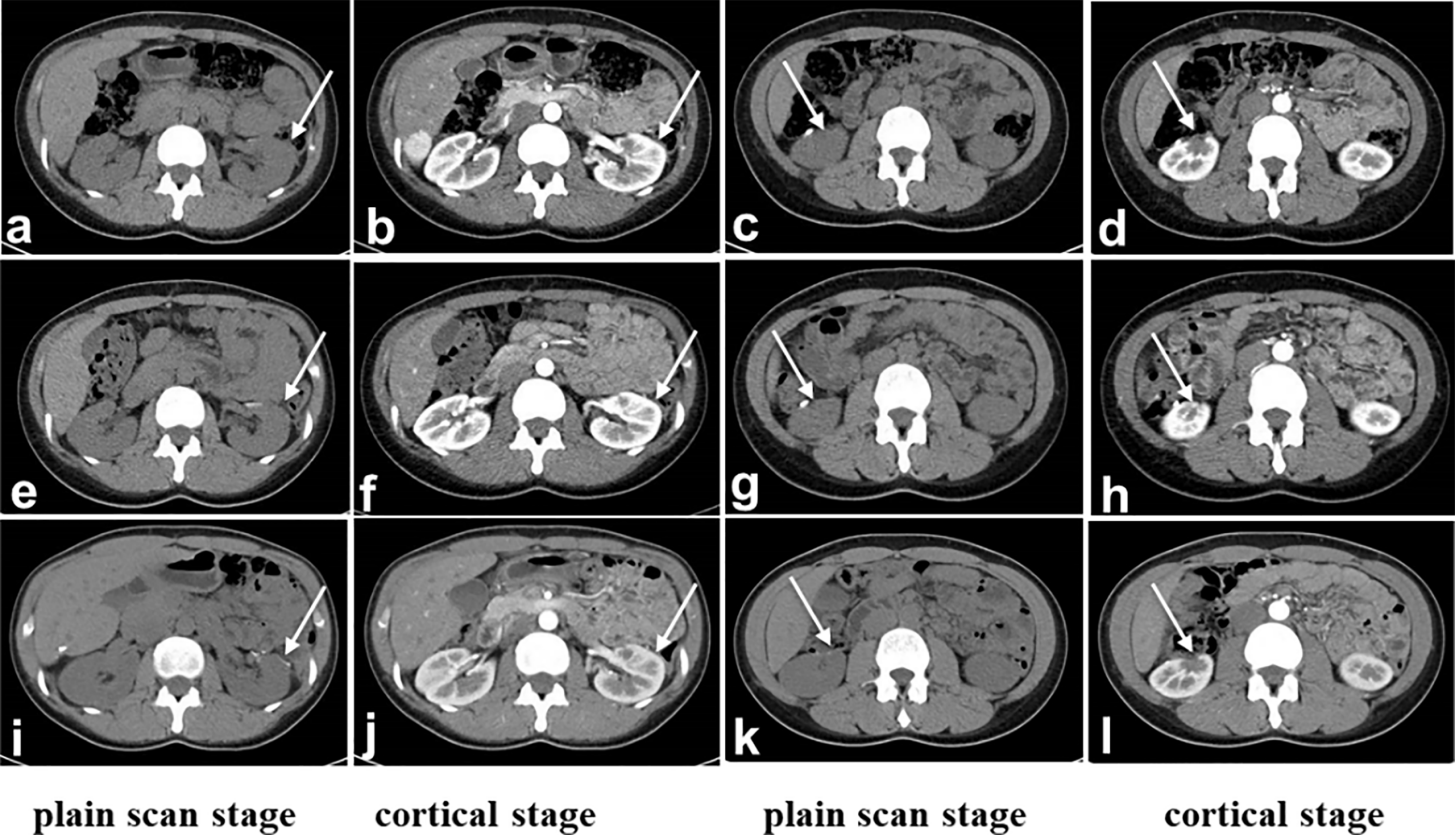
CT after surgery. **(A-D)**: Third month after surgery, **(E-H)**: 6 months after surgery, **(I-L)**: 24th month after surgery. The margins of the upper left kidney **(A, B, E, F, I, J)** and lower right kidney **(C, D, G, H, K, L)** were irregular. The perirenal space of the bilateral kidneys was clear, and the shape and size were normal. There was no obvious enlarged lymph node shadow in the retroperitoneum. **(A, E, I)**: Plain scan stage. **(B, F, J)**: Cortical stage. **(C, G, K)**: parenchymal stage. **(D, H, L)**: Renal pelvis stage. The white arrow indicates status of partial nephrectomy.

We report a 25-year-old woman with bilateral renal tumors detected by ultrasound during a routine checkup. She had no lumbago, hematuria, fever, and other symptoms as well as no significant change in body weight. Physical examinations (PE) showed the no positive signs, and skin mucous membrane was normal without any nodule. Color ultrasonography results showed that there was an abnormal echo area (7 mm × 6 mm) in uterine parenchyma, with a clear boundary and a regular shape, mainly with low echo, and without blood flow signal, considering the possibility of uterine leiomyomas. Computerized tomography (CT) results showed a soft tissue mass (36 mm × 37 mm × 37 mm) with calcification, and uneven enhancement was found in the parenchyma of the left kidney, which protruded into the renal capsule and was closed to the spleen and pancreas. A nodular soft tissue (12 mm × 12 mm × 13 mm) was also found in the lower pole of the right kidney, which extended outward the renal capsule and was closed to the intestinal tract; the TNM stage was T3aN0M0 (**Figure 1**). For the treatment, the patient underwent robot-assisted laparoscopic left partial nephrectomy and 3 months after first surgery underwent laparoscopic partial right nephrectomy. Both operations completely cut the tumor at about 1 cm at the edge of the tumor. The gross of the tumor appeared as moderate hardness, 37 mm of the left kidney tumor and 12 mm of the right kidney tumor, with grayish-white cut surface, and no cancer cells were seen at the tumor margins. Postoperative pathology revealed that the tumor cells were arranged into tubes or sacs with abundant eosinophilic cytoplasm and obvious nucleolus. No obvious mitosis was observed. Intercellular blood vessels were increased, dilated, and congested (**Figure 2**). Immunohistochemical staining showed the following results: CK7 (+),Vim (+), SDHB (+), CK (–), CD10 (-), AMACR (-), CD117 (-), CAIX (-), P63 (-), Ki67 (<1%), TFE3(-). FH staining of the tumor tissue revealed the loss of FH expression. Pathological results after surgery indicated the following: considering the high possibility of chromophobe renal cell carcinoma, the patient was treated with anti-infection, nutritional support, and bleeding prevention, and the patient was discharged on the 9th postoperative day. In order to confirm the diagnosis and provide the basis for the follow-up treatment, we performed genetic testing on the patient’s tumor tissue and found that the patient carried a heterozygous mutation in the *FH* gene. Combined with the patient’s medical history, microstructure, and immunohistochemical staining of tumor tissue and genetic test results, the pathological reports of tumor tissue after two operations concluded HLRCC-associated RCC. Then, we performed genetic testing on her parents about *FH*-c.737delA (p.Gln246ArgfsX10) and found that her father does not have the *FH* gene mutation and her mother who had a heterozygous mutation in the *FH* gene did not have any HLRCC-associated diseases. After operation, the patient was injected with interferon 9 × 10^6^ IU per day for 3 months and nivolumab OPDIVD 100 mg/time biweekly for 12 months as a preventative treatment against tumor recurrence, and no side effect was shown. Immunotherapy ended when no recurrence or metastasis of the cancer was detected by CT 1 year after the operation. The red and white cell counts and the renal function index were in the normal range during and after the treatment (**Figure 3** and **Supplementary Image 1**). CT was performed at the third, sixth, and 24th months after the second operation (**Figure 4**). She was pregnant at the 28th month after the second surgery and gave birth to her child at the 38th month after the second surgery. During pregnancy, she had no clinical manifestations of renal cancers; till now, there was no tumor progression.

## Discussion

HLRCC-associated RCC is an autosomal dominant genetic RCC caused by germline mutations of the *FH* gene which is an enzyme that catalyzes the transformation of fumaric acid into malic acid in the Krebs cycle (6, 7). Mutation of the *FH* gene leads to the accumulation of fumaric acid which resulted in overexpression of oncogene *HIFα*, thereby promoting the formation of uterine fibroids, cutaneous leiomyomas, and renal cancers (8, 9). Unlike other types of hereditary renal cell carcinoma, HLRCC-associated RCC is usually unilateral, early-onset, aggressive, and rapidly progressing, and many patients already have metastasis by the time of diagnosis (10, 11).

Immunotherapy has emerged as a new direction of cancer treatment for patients with RCC. Some studies have shown that ICIs (immune checkpoint inhibitors) are a new treatment option of RCC. Several cases of papillary RCC were successfully treated with nivolumab (12, 13). A case report recently showed the achievement of complete response in a patient with HLRCC-associated RCC of ICI combination treatment (nivolumab plus ipilimumab) after 31 weeks (14). In another report, PD-1 has been shown to improve the prognosis of patients with HLRCC (15). Based on the above research, the patient with HLRCC-associated RCC was injected with nivolumab postoperation for prevention of RCC recurrence. However, the adjuvant therapy provided to this patient is not supported by treatment guidelines and is the not standard of care. The adjuvant treatment provided could be toxic with long-term side effects; we think that the efficiency of this treatment strategy needs further investigation in clinic.

Prompt excision of HLRCC-associated kidney tumors is critical for preventing metastasis (16). There are neither reports of pregnancy in patients after partial nephrectomy for HLRCC-associated RCC nor reports of pregnancy in patients after twice partial nephrectomy for bilateral kidneys with HLRCC-associated RCC. This is the first reported case of safe pregnancy in a patient with bilateral HLRCC-associated RCC who underwent two partial nephrectomies. Although the patient had uterine fibroids, she had no related gynecological symptoms and the fertility was not affected. After twice partial nephrectomies and a preventative treatment with interferon and nivolumab, she was pregnant at the 28th month after the second surgery. During pregnancy, she had no clinical manifestations of renal cancers and color ultrasound on the urinary system showed no significant change. According to previous reports, if a patient carries *FH* gene mutation, early reproduction should be considered because the patient is at high risk of early hysterectomy and infertility (17–19). However, this case provides a reference for pregnancy in patients carrying *FH* gene mutation, even though she had uterine fibroids and bilateral HLRCC-associated RCC.

When *FH* gene mutation was tested, a complete family history should be investigated (20). Moreover, doctors must be aware of this rare genetic disorder and recommend that patients and their families undergo gene sequencing to confirm the presence of the *FH* gene mutation (3). If there is an *FH* gene mutation, genetic diagnosis should be made to guide fertility and family health supervision which is the key to preventing metastasis of HLRCC-associated RCC.

## Conclusion

This is a clinically significant case, as it provides a reference for pregnancy in patients undergoing partial nephrectomy for bilateral kidneys with HLRCC-associated RCC and may indicate an effective approach to prevent tumor recurrence by nivolumab in patients with HLRCC-associated RCC.

## Data availability statement

The original contributions presented in the study are included in the article/**Supplementary material**. Further inquiries can be directed to the corresponding author.

## Ethics statement

The studies involving human participants were reviewed and approved by Medical Ethics Committee of Chongqing Medical University. The patients’ data were anonymized and de-identified. Written informed consent was obtained from patients or their authorized trustees.

## Author contributions

KD: drafting of the manuscript, acquisition of data; WJ: drafting of the manuscript; SC: acquisition of data; SL: writing—review and editing; SD: writing—review and editing; DW: supervision, funding acquisition. All authors agree to be accountable for the content of the work.

## Funding

This work was supported by the Chongqing Science and Technology Commission (cstc2017shms-zdyf0319, 2018jstg008, cstc2016shms-ztzx130001, and 2020jstg018).

## Conflict of interest

The authors declare that the research was conducted in the absence of any commercial or financial relationships that could be construed as a potential conflict of interest.

## Publisher’s note

All claims expressed in this article are solely those of the authors and do not necessarily represent those of their affiliated organizations, or those of the publisher, the editors and the reviewers. Any product that may be evaluated in this article, or claim that may be made by its manufacturer, is not guaranteed or endorsed by the publisher.

## Supplementary material

Supplementary Image 1 Red and White cells count after surgery. **(A)**:Changes of red cells count after surgery, **(B)**:Changes of white cells count after surgery. The levels of red and white cells count after surgery follow-up were in the normal range.
