# Supplementary figures and images for: Case report: Going through pregnancy safely after twice partial nephrectomy for bilateral kidneys with HLRCC-associated RCC
Source: Front Oncol. 2022 Oct 18;12:932996. doi: 10.3389/fonc.2022.932996 (PMC9623055; doi:10.3389/fonc.2022.932996)

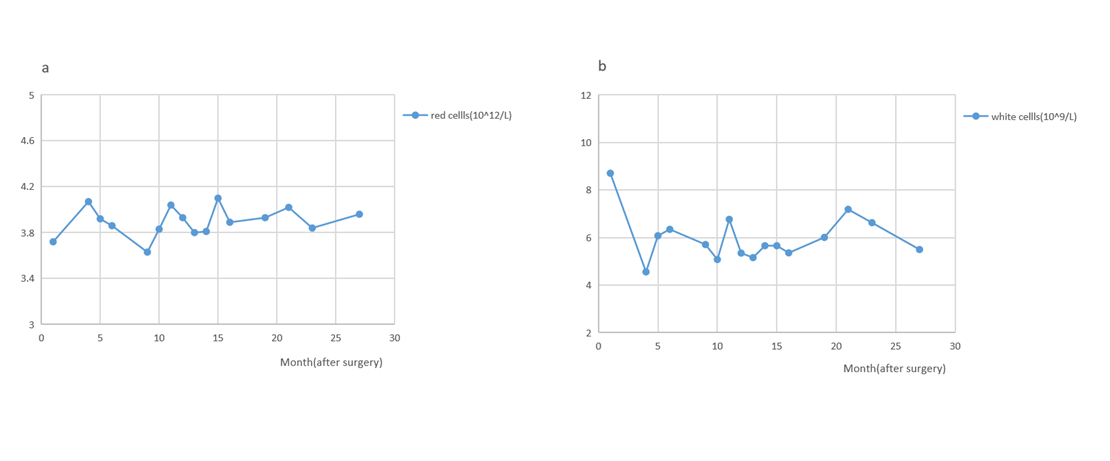

Supplement: Supplementary Image 1 — Red and White cells count after surgery. (A):Changes of red cells count after surgery, (B):Changes of white cells count after surgery. The levels of red and white cells count after surgery follow-up were in the normal range. [file Image_1.jpeg]
